# Hearing Status, Social Engagement, and Longitudinal Changes in Cognition: Results From the Mexican Health and Aging Study

**DOI:** 10.1155/jare/7546410

**Published:** 2026-09-02

**Authors:** Chengming Han, Joey M. Saavedra, Brian Downer, Rebeca Wong

**Affiliations:** ^1^ Independent Researcher, Calgary, Canada; ^2^ Independent Researcher, Beijing, China; ^3^ University of Texas Medical Branch at Galveston, Galveston, Texas, USA, utmb.edu; ^4^ University of Texas Health Science Center at San Antonio, San Antonio, Texas, USA, uthscsa.edu

**Keywords:** cognition, hearing, Mexico, social engagement

## Abstract

**Objectives:**

This study explored the mediation and moderation effects of social engagement on the relationship between hearing status and cognitive function among Mexican adults aged ≥ 50 years across a six‐year period (2015–2021).

**Methods:**

Data were drawn from the nationally representative Mexican Health and Aging Study (MHAS). Social engagement was calculated by summing the number of social activities and frequencies undertaken in the past year. A global measure of cognitive function was derived by summing *z*‐scores of verbal memory, orientation, verbal fluency, visual scanning, figure copy, and figure recall. Multilevel generalized models with lagged predictors were used to assess the mediation and moderation effects of social engagement on the association between self‐reported hearing status and cognitive function over time.

**Results:**

There were 10,561 people in the analytic sample. Greater social engagement was independently associated with better cognitive function over time. Better hearing status was associated with higher cognitive functioning in the following survey wave. Fair or better hearing was significantly associated with increased social engagement. The association was attenuated when covariates were adjusted. The association between hearing difficulty and cognitive function varied across levels of social engagement.

**Discussion:**

The mediation and moderation effects of social engagement on the association between hearing difficulty and cognitive function were affected by the population’s socioeconomic status among middle‐aged and older adults in Mexico. Future research should investigate other unknown mediators between hearing difficulty and cognitive health of middle‐aged and older adults in low‐ and middle‐income countries.

## 1. Introduction

Aging is associated with heterogeneous losses in cognitive abilities, including learning, reasoning, and memory [1, 2]. Exaggerated declines in cognition are associated with an increased risk of mild cognitive impairment (MCI), a transitional state between normal cognitive aging and dementia (e.g., Alzheimer’s disease), characterized by lower‐than‐expected cognitive performance while maintaining independence in daily activities [3, 4]. The prevalence of MCI among adults aged 50 years and older in the United States is approximately 22%, with Hispanic older adults 1.4 times more likely to have MCI compared to non‐Hispanic White older adults [5]. While there is considerable epidemiological research on the prevalence and risk factors of cognitive impairment and cognitive decline among US Hispanic populations, less is known about older adults in Mexico, and the age‐related antecedents of cognitive decline in Mexican older adults within the unique socioenvironmental context of Mexico are poorly understood.

As many as 1 in 3 middle‐aged and older adults in Mexico report experiencing hearing loss or poor hearing [6, 7]. Evidence suggests that age‐associated hearing loss influences cognitive decline in later life [8, 9]. For instance, a 2018 meta‐analysis of nine cohort studies with approximately 8000 older adults found that hearing loss was significantly associated with poorer global cognition [9]. Similarly, a 2024 meta‐analysis of 18 observational studies with approximately 19,000 older adults found that each decrement in objectively assessed hearing was associated with an average reduction of 0.13 standard deviations in global cognition scores [8]. However, most of these studies were conducted in countries with advanced economies and cultural contexts that differ from those in Mexico, potentially limiting generalizability to older adults in this population.

Engaging in social activities can help protect against cognitive decline and dementia [10], but hearing loss can severely limit older adults’ ability to participate in these activities [11]. Thus, reduced social engagement may serve as a mechanism through which hearing loss contributes to cognitive decline. Hearing difficulties increase the cognitive load needed to participate in social settings, making social interactions more challenging [12]. Individuals with hearing loss and hearing difficulties are also more likely to decrease their social activities, have smaller social networks [13–15], and experience increased depressive symptoms [16]. Prior evidence in older Mexicans suggests that perceived hearing loss was significantly associated with increased loneliness [17].

Mexico has experienced rapid population aging over the past decades. However, compared to high‐income aging countries, Mexico faces unique socioeconomic challenges that make older adults particularly susceptible to adverse outcomes. For instance, Mexico lacks a nationwide social security safety net, has a much smaller public pension system that covers fewer than half of public sector workers, and has high poverty rates and insufficient medical resources, all of which coalesce to disproportionately affect outcomes in the aging population [18, 19]. Mexican society is also characterized by a heavy reliance upon familial connections across the lifespan, with the nuclear network serving as the most reliable source of financial and social support for older adults [20]. Indeed, social engagement in Mexico is largely family‐based. Being accompanied by family members is an important form of social participation for both Mexican women and men. The number of siblings, household size, family networks, and encouragement from family members to participate in outdoor social activities also promote social engagement [21, 22]. Compared to the United States and the United Kingdom, the rate of social participation in Mexico is substantially lower [23].

While considerable evidence exists regarding the relationship between hearing status, social engagement, and cognitive functioning, the potential mediating role of social activities on the longitudinal association between hearing status and cognitive decline in older adults in Mexico remains unexplored. Therefore, we evaluated the role of social activities in the relationship between hearing status and cognitive function using longitudinal data from the Mexican Health and Aging Study (MHAS). Furthermore, few studies have examined the possible protective effects of social activities on hearing status, as well as the moderate role of social activities in the association between hearing difficulties and cognitive function.

### 1.1. Conceptual Framework

**FIGURE 1:**
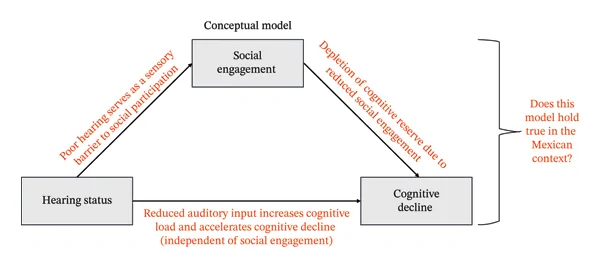
The conceptual framework for the mediation effects of social engagement.

We anchor our analyses to a biopsychosocial framework that integrates perspectives from social gerontology, cognitive neuroscience, and sensory epidemiology to explain the hypothesized relationships between hearing status, social engagement, and cognition (Figure 1). Age‐related hearing loss contributes to auditory deprivation, and the subsequent reduction in sensory input may promote cognitive decline by hampering neuroplasticity [24] and increasing cognitive load [25]. Age‐related hearing loss serves as a sensory barrier to participation in activities such as group recreation or religious services [12], where communication difficulties, perceived stigma, and negative self‐perception increase the likelihood of withdrawal from socially stimulating activities [11]. Losses to a person’s social network contribute to social isolation and loneliness [26], and the chronic absence of cognitive stimulation may accelerate the compression of cognitive reserve [27]. The cognitive reserve theory posits that sustained social engagement builds neural reserve that may buffer the brain against the cognitive consequences of sensory impairment [28]. It is therefore possible that older adults who maintain higher levels of social engagement over time experience attenuated cognitive decline even in the presence of hearing difficulty.

We examined three research questions: First, is hearing difficulty longitudinally associated with cognitive decline among adults aged 50 and older in Mexico? Second, is hearing difficulty associated with decreased social activity over time, which in turn negatively influences the cognitive functioning of middle‐aged and older adults in Mexico? Third, does the association between hearing difficulties and cognitive decline vary according to the level of social engagement over time among older adults? Accordingly, we proposed three hypotheses as follows:

 Hypothesis 1. Better hearing status will be associated with better cognitive function among middle‐aged and older adults in Mexico.
 Hypothesis 2. Better hearing status may be associated with more social engagement among Mexicans.
 Hypothesis 3. As previous studies have demonstrated positive effects of social engagement on cognitive function, levels of social engagement may influence the association between hearing status and cognition in Mexico.

The results of this analysis provide novel insights into social behaviors relevant to Mexican older adults, which can be promoted to mitigate the detrimental effects of age‐related hearing loss on cognitive health.

## 2. Methods

### 2.1. Data and Sample

Data for this study came from the MHAS, an ongoing, nationally representative cohort study of adults aged 50 and older in Mexico. The MHAS began in 2001 with a sample of approximately 15,000 participants, with follow‐up interviews completed in 2003, 2012, 2015, 2018, and 2021. In 2012, a new sample of nearly 5900 participants aged 50–59 was added to maintain national representativeness. Most participants completed a direct (i.e., face‐to‐face) interview that included questions on demographic and socioeconomic characteristics, household composition and family structure, health insurance and healthcare utilization, physical and daily functioning, and assistance provided or received for daily activities. Approximately 5% of participants required a proxy interview at each wave. The MHAS study protocol and instruments were approved by the Institutional Review Board or Ethics Committee of the INEGI in Mexico, the University of Texas Medical Branch, and the Instituto Nacional de Salud Pública (INSP) in Mexico [29, 30].

**FIGURE A1:**
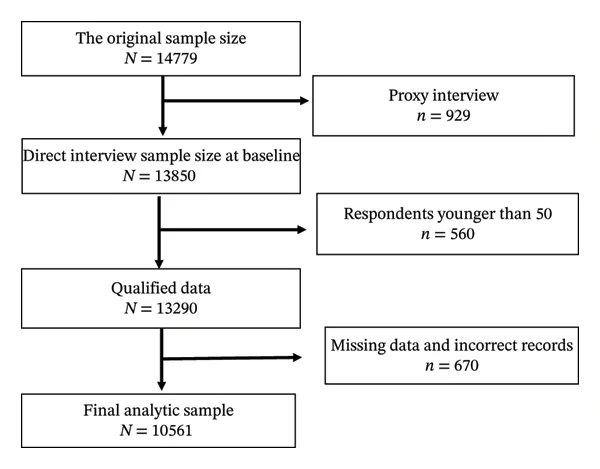
Election of analytical samples.

This study used data from the 2015, 2018, and 2021 waves of the MHAS. We selected the 2015 wave as the baseline because it was the first interview in which all participants were asked about their hearing status (see Figure A1 in the Appendix for the sample selection criteria). First, due to the fact that proxy interviews did not have cognitive function test results, we selected participants who completed the survey independently, excluding those who relied on proxy assistance. Second, we selected participants aged 50 or older in the 2015 wave. Third, we selected participants with complete information on hearing status, social activities, demographic characteristics, and cognitive function. The final analytic sample included 10,561 participants. The characteristics of missing data are presented in Appendix Tables A1 (baseline) and A2 (person‐years). Of those excluded from the sample, participants were generally older, had lower cognitive function, had poorer hearing, and reported lower social engagement. As such, the final analytic sample is likely healthier than the overall MHAS cohort.

### 2.2. Dependent Variable

Since the 2015 baseline wave, participants who received a direct interview completed a set of seven cognitive tasks, including immediate word recall, delayed word recall, orientation, visual scanning, verbal fluency (animal naming), figure copy, and figure recall. The immediate and delayed word list recall task requires participants to repeat a list of eight words back to the interviewer. After a delay of several minutes, participants are asked to recall as many words as possible from the list, with a score range of 0–8 points. The orientation task requires participants to correctly state the current day, month, and year. One point is awarded for each correct response, with a score range of 0–3 points. For the visual scanning task, participants are given one minute to circle a target stimulus (60 items) in a visual array, with a score range of 0–60 points. The verbal fluency (animal naming) task requires participants to name as many animals as they can in 1 min. Finally, in the figure copy and recall task, participants are shown a figure composed of intersecting lines and shapes, which they must copy. After a delay of several minutes, participants are asked to draw the figure from memory, with a score range of 0–6 points.

Our dependent variable was a summary measure of global cognition. For each interview wave, we first calculated a baseline‐centered z‐score for each cognitive task by standardizing each cognitive task score into z‐scores using the baseline mean and standard deviation of the analytic sample. We then summed the seven z‐scores to create a composite of global cognition for each observation wave [31, 32]. To adjust for the different weights of the cognitive task, we divided the final z‐score by seven [31]. The z‐score range of global cognition across all waves was −2.802 to 1.678, with higher scores indicating better cognitive functioning. Detailed information on the range of each cognitive task is presented in Appendix Table A3.

### 2.3. Independent Variables

The key independent variables included hearing status, hearing aid use, and social engagement. All independent variables were coded as time‐varying. For hearing status, participants were asked to rate their hearing/auditory range on the following scale: excellent, very good, good, fair, or poor. We coded poor hearing as 1 and excellent hearing as 5. For those reporting the use of a hearing aid or auditory device, hearing status reflected their hearing ability while wearing their assistive device. We also included hearing aid use as a binary covariate (1 = yes, 0 = no) because some participants wore their hearing aids during the survey.

Social engagement was characterized using self‐reported responses to survey questions about participation in any of the following in the preceding year: Volunteering/supporting an organization without pay/rewards, attending a training course, attending a sporting club, playing games, and communicating with relatives/friends via phone/internet. The number of times a participant reported engaging in these activities (i.e., activity frequency) was converted to deciles. This value was then multiplied by the total number of different activities a participant reported undertaking, providing a final value ranging from 0 to 50 (with a higher value indicating greater social engagement). Our operationalization of social engagement was consistent with prior aging research that conceptualizes social participation as involvement in interpersonal, community, leisure, and cognitively stimulating activities that were found to be positively associated with better cognitive functions [33, 34]. Furthermore, since previous research shows that leisure activities such as reading a book or crafting are favorably associated with cognition in older adulthood [35], we also examined the effects of solo activities, which allow us to evaluate the robustness of our approach across differing definitions of social engagement. We created a second metric of social engagement that included all types of activities (e.g., all the activities in the first metric plus the following: Caring for children under 12 years, caring for an adult with disabilities, crafting or sewing, reading books, watching TV, playing puzzles, gardening, and interactive activities).

### 2.4. Covariates

Covariates included education (coded as 0 for formal education, 1 for elementary school or below, and 2 for greater than elementary school), income, residence, age at baseline (years), and gender (male or female). Educational attainment is typically low among older adult Mexicans because the accessibility to elementary school was extremely limited until the 1950s [36], a time when the majority of the analytic sample was of schooling age. Income was coded by the tertile: High, medium, and low. Residence was coded as urban (community size > 100,000) and rural (community size < 100,000).

### 2.5. Statistical Analysis

Descriptive results presented characteristics of the whole sample and were stratified by hearing status. A series of multilevel generalized mixed models was employed to estimate the association between hearing status and cognitive decline. We estimated both mixed models with a random intercept and a random slope. The maximum likelihood test showed that the model with a random intercept was not significantly nested within the model with a random slope, and the random slope is close to zero (1.03e − 15). We selected the model with a random intercept as the final model to estimate the effects on cognitive function. Given the right‐skewed distribution of social engagement, we used a multilevel negative binomial model to estimate the effects of hearing status on social engagement. Furthermore, to estimate the causal effects of hearing status on social engagement and cognitive function, we included lagged effects in the model. The chain of Model 1 regressed the global cognition on hearing status at Time‐1, without adjusting for social engagement and covariates. Model 2 examined the effects of hearing status at Time‐1 and social engagement without adjusting for covariates. Model 3 assessed the effects of hearing status at Time‐2, social engagement at Time −1, and global cognition, without adjusting for covariates. Models 4–6 repeated the estimations from Models 1–3 and adjusted for all covariates in each model. Models 7–9 estimated the mediation effects of all activities. To evaluate the moderation effect of social engagement on the relationship between hearing status and cognitive functioning, interaction effects between hearing status and levels of social engagement were tested. All estimations were conducted in Stata 18.0, using two‐sided tests with statistical significance defined as *p* < 0.05. Sampling weight was not used.

## 3. Results

Table 1 illustrates the characteristics of the whole sample and of participants with different hearing statuses. The average age at baseline was 65.87 (SD = 8.99) years; 59% of participants were female, and the average number of reported social engagement (adjusted for frequency) was 8.21 (SD = 8.88). At baseline, around 34% of participants reported fair/poor hearing, while 1.72% reported using a hearing aid. Participants with poor or fair hearing are older, and they exhibited lower cognitive function (poor hearing: −0.34; fair hearing: −0.02). Their social engagement is lower than average (poor hearing: 5.09 and fair hearing: 7.27 vs. whole sample: 8.21). On the other hand, participants with good, very good, or excellent hearing status showed higher cognitive function, more social engagement, higher education, higher proportion of urban residence, higher proportion of women, and younger age.

**TABLE 1:** Descriptive results of the final sample at baseline (*N* = 10,145)^a^.

Variable	Whole sample	Poor	Fair	Good	Very good	Excellent
Observations	10,145	381	3076	5264	956	468
Cognition (*z*‐score, range: −2.33, 1.68)	0.05 (0.64)^b^	−0.34 (0.75)	−0.02 (0.61)	0.08 (0.63)	0.18 (0.61)	0.24 (0.60)
Hearing status						
Poor	3.76%					
Fair	30.32%					
Good	51.89%					
Very good	9.42%					
Excellent	4.61%					
Using a hearing aid	1.72%	6.04%	2.15%	1.22%	1.57%	1.28%
Social engagement (0–50)	8.21 (8.80)	5.09 (6.87)	7.27 (7.80)	8.48 (9.02)	9.74 (9.73)	10.81 (10.32)
Education						
No education	14.65%	27.30%	15.80%	14.19%	11.19%	8.97%
< 6 years	30.51%	37.01%	35.40%	28.59%	26.36%	23.08%
≥ 6 years	54.84%	35.70%	48.80%	57.22%	62.45%	67.95%
Income						
Low	32.25%	27.30%	30.92%	33.47%	31.80%	32.26%
Medium	33.87%	47.77%	36.67%	32.64%	27.82%	30.34%
High	33.88%	24.93%	32.41%	33.89%	40.38%	37.39%
Urban residence	56.61%	49.61%	54.49%	56.38%	62.55%	66.67%
Sex						
Man	41.50%	49.61%	45.68%	38.58%	38.81%	45.73%
Women	58.50%	50.39%	54.32%	61.42%	61.19%	54.27%
Age at baseline	65.87 (8.99)	72.78 (9.62)	67.38 (9.20)	65.10 (8.62)	64.15 (8.33)	62.51 (8.00)

^a^The sample size at baseline is different from the final sample size in the mixed models.

^b^Mean (SD).

Table 2 presents the lagged regression results across waves 2015–2021. Model 1 shows a significant direct effect of lagged hearing status on global cognition; in other words, participants with fair hearing showed higher global cognition than those with poor hearing for the next wave (*b* = 0.033, SE = 0.015, *p* = 0.024). As we divided the final global cognitive z‐score by seven cognitive tasks, the difference in raw cognitive score (0–131) was as follows: 0.033 ∗ 7 ∗ SD = 0.033 ∗ 7 ∗ 22.934 = 5.298. The magnitude increased for participants with good (*b* = 0.045, SE = 0.015, *p* = 0.002), very good (*b* = 0.056, SE = 0.016, *p* = 0.001), and excellent hearing (*b* = 0.065, SE = 0.018, *p* = 0.000). In other words, compared with participants who had poor hearing, those with fair to excellent hearing scored 5–10 points higher on the global cognition measure. Model 2 shows that compared to those with poor hearing, participants with fair or better hearing displayed slightly more social engagement at the next measured time. For instance, participants with fair hearing status showed more social engagement than those with poor hearing status in the next wave (*b*_fair_ = 0.092, SE_fair_ = 0.033, *p* = 0.005), while the effect size is slight. Model 3 measures the chain of hearing status at the first measurement time, social engagement at the second measurement time, and global cognition at the third measurement time. The results showed that participants with excellent hearing showed a higher (*b* = 0.055, SE = 0.019, *p* = 0.005) unit higher standard global cognition than those with poor hearing, and those with very good hearing exhibited a higher (*b* = 0.043, SE = 0.018, *p* = 0.014) unit higher standard global cognition than those with poor hearing. Social engagement at the last wave predicts higher standardized global cognition (*b* = 0.005, SE = 0.000). This means one unit higher social engagement contributes to around one raw cognitive score (0.005 ∗ 7 ∗ 22.934).

**TABLE 2:** Mixed‐effects models of lagged hearing and activity engagement predicting cognitive function.

	Unadjusted	Social engagement		Adjusted	Social engagement		All activities	
	Hearing ⟶ cognition	Hearing ⟶ social engagement	Hearing and social engagement ⟶ cognition	Hearing ⟶ cognition	Hearing⟶ social engagement	Hearing and social engagement ⟶ cognition	Hearing ⟶ social engagement	Hearing and social engagement ⟶ cognition
*Hearing status at t-1 (ref = poor hearing)*								
Fair	0.033^∗^ ^a^	0.092^∗∗^		0.024	0.002		0.010	
	(0.015)	(0.033)		(0.014)	(0.033)		(0.028)	
	0.024^b^	0.005		0.096	0.953		0.730	

Good	0.045^∗∗^	0.150^∗∗∗^		0.029^∗^	0.047		0.036	
	(0.015)	(0.033)		(0.014)	(0.033)		(0.028)	
	0.002	0.000		0.041	0.151		0.204	

Very good	0.056^∗∗∗^	0.119^∗∗^		0.037^∗^	0.006		0.041	
	(0.016)	(0.036)		(0.016)	(0.036)		(0.031)	
	0.001	0.001		0.021	0.879		0.188	

Excellent	0.065^∗∗∗^	0.092^∗^		0.043^∗^	−0.006		0.055	
	(0.018)	(0.040)		(0.017)	(0.040)		(0.034)	
	0.000	0.021		0.014	0.886		0.112	

Using hearing aid(s)	−0.007	−0.105^∗^	−0.004	−0.029	−0.052	−0.033	−0.053	−0.033
	(0.020)	(0.045)	(0.022)	(0.019)	(0.044)	(0.021)	(0.038)	(0.021)
	0.073	0.018	0.849	0.132	0.242	0.125	0.150	0.126

*Hearing status at t-2 (ref = poor hearing)*								
Fair			0.023			0.022		0.019
			(0.016)			(0.015)		(0.015)
			0.152			0.154		0.225

Good			0.027			0.018		0.014
			(0.016)			(0.015)		(0.015)
			0.085			0.247		0.346

Very good			0.043^∗^			0.027		0.023
			(0.018)			(0.017)		(0.017)
			0.014			0.111		0.176

Excellent			0.055^∗∗^			0.034		0.029
			(0.019)			(0.019)		(0.019)
			0.005			0.069		0.128

Social engagement or activity engagement at t‐1			0.005^∗∗∗^			0.003^∗∗∗^		0.002^∗∗∗^
			(0.000)			(0.000)		(0.000)
			0.000			0.000		0.000

Female	−0.027^∗∗^	0.100^∗∗∗^	−0.026^∗^	0.020^∗^	0.176^∗∗∗^	0.018^∗^	0.327^∗∗∗^	0.012
	(0.010)	(0.023)	(0.011)	(0.009)	(0.021)	(0.009)	(0.014)	(0.009)
	0.008	0.000	0.013	0.019	0.000	0.046	0.000	0.209

Age at baseline	−0.033^∗∗∗^	−0.035^∗∗∗^	−0.033^∗∗∗^	−0.025^∗∗∗^	−0.017^∗∗∗^	−0.026^∗∗∗^	−0.014^∗∗∗^	−0.026^∗∗∗^
	(0.001)	(0.001)	(0.001)	(0.001)	(0.001)	(0.001)	(0.001)	(0.001)
	0.000	0.000	0.000	0.000	0.000	0.000	0.000	0.000

*Education (ref = no education)*							(0.037)	(0.021)
Incomplete elementary school				0.346^∗∗∗^	0.234^∗∗∗^	0.353^∗∗∗^	0.329^∗∗∗^	0.347^∗∗∗^
				(0.014)	(0.033)	(0.014)	(0.022)	(0.014)
				0.000	0.000	0.000	0.000	0.000

Complete elementary school and upper				0.672^∗∗∗^	0.669^∗∗∗^	0.670^∗∗∗^	0.698^∗∗∗^	0.659^∗∗∗^
				(0.013)	(0.032)	(0.014)	(0.021)	(0.014)
				0.000	0.000	0.000	0.000	0.000

*Income (ref = low)*								
Medium income				0.016^∗^	0.093^∗∗∗^	0.012	0.087^∗∗∗^	0.012
				(0.007)	(0.015)	(0.007)	(0.013)	(0.007)
				0.019	0.000	0.107	0.000	0.093

High income				0.071^∗∗∗^	0.241^∗∗∗^	0.070^∗∗∗^	0.203^∗∗∗^	0.071^∗∗∗^
				(0.007)	(0.017)	(0.008)	(0.014)	(0.008)
				0.000	0.000	0.000	0.000	0.000

Urban residence				0.121^∗∗∗^	0.168^∗∗∗^	0.111^∗∗∗^	0.202^∗∗∗^	0.106^∗∗∗^
				(0.009)	(0.021)	(0.009)	(0.014)	(0.009)
				0.000	0.000	0.000	0.000	0.000

Constant	2.176^∗∗∗^	3.663^∗∗∗^	2.140^∗∗∗^	1.085^∗∗∗^	2.014^∗∗∗^	1.089^∗∗∗^	3.149^∗∗∗^	1.080^∗∗∗^
	(0.042)	(0.093)	(0.045)	(0.041)	(0.097)	(0.043)	(0.067)	(0.043)
	0.000	0.000	0.000	0.000	0.000	0.000	0.000	0.000

*Random effects*								
SE of random intercept (ln)	−0.788^∗∗∗^		−0.808^∗∗∗^	−1.009^∗∗∗^		−1.017^∗∗∗^		−1.027^∗∗∗^
	(0.009)		(0.010)	(0.010)		(0.011)		(0.011)
	0.000		0.000	0.000		0.000		0.000

SE of residual error (ln)	−1.217^∗∗∗^		−1.204^∗∗∗^	−1.213^∗∗∗^		−1.205^∗∗∗^		−1.203^∗∗∗^
	(0.007)		(0.008)	(0.007)		(0.008)		(0.008)
	0.000		0.000	0.000		0.000		0.000

Overdispersion (ln)		−1.161^∗∗∗^			−1.460^∗∗∗^		−1.112^∗∗∗^	
		(0.021)			(0.023)		(0.016)	
		0.000			0.000		0.000	

SE of the random intercept		1.049^∗∗∗^			0.729^∗∗∗^		0.233^∗∗∗^	
		(0.022)			(0.017)		(0.007)	
		0.000			0.000		0.000	

Person‐years	22,119	25,929	18,567	21,851	21,851	18,333	21,850	18,332

Number of observations	10,100	10,953	9302	9995	9995	9200	9995	9199

Log‐likelihood	−13.408	−75.667	−11.504	−11.585	−65.243	−9909	−91.944	−9877

Chi‐square	3348	899.9	3362	8568	1657	7930	3538	8087

^a^Coefficient, standard errors in parentheses.

^b^ *p* value.

^∗^ *p* < 0.05.

^∗∗^ *p* < 0.01.

^∗∗∗^ *p* < 0.001.

After adjusting for covariates in Models 4‐6, the association between hearing status and global cognition at the next wave remained significant, though the magnitude of this association was lower compared to that of the unadjusted model (*b*_good_ = 0.029, SE_good_ = 0.014, *p* = 0.041; *b*_very good_ = 0.037, SE_very good_ = 0.016, *p* = 0.021; *b*_excellent_ = 0.043, SE_excellent_ = 0.017, *p* = 0.014). Hearing status at the earlier wave showed no significant effects on social engagement after adjusting for covariates. Hearing at the first measurement time showed no significant effects on global cognition after adjusting for social engagement at the second measurement time and covariates. Social engagement at the previous measurement time had significant effects (*b* = 0.003, SE = 0.000, *p* = 0.000) on global cognition, adjusting for all covariates. Moreover, we differentiated between social engagement likely to be performed with others and those that could be performed alone, such as playing puzzles, reading, gardening, watching TV, performing home maintenance or repairs, and sewing or partaking in other crafts. The results of Models 7 and 8 revealed a similar pattern between hearing status and activities that include solitary leisure activities, as well as the association between hearing status and global cognition.

The moderation analysis first revealed a significant association between different levels of social engagement and hearing status (Table 3). Participants with fair/good/very good hearing status showed lower global cognition with a higher level of social engagement (*b*_fair_ = −0.005, SE_fair_ = 0.002, *p* = 0.019; *b*_good_ = −0.006, SE_good_ = 0.002, *p* = 0.003; *b*_very good_ = −0.005, SE_very good_ = 0.002, *p* = 0.011) (Table 3). It is possible that the protective association of greater social engagement and cognitive functioning is stronger for participants with poor hearing, while participants with good hearing may not experience as many cognitive benefits from high levels of social engagement as people with poor hearing. When all activities were included, no significant moderation effects were detected. It is possible that other activities such as watching TV or reading a book are not related to hearing status.

**TABLE 3:** Moderation effects of social engagement on the association between hearing status and cognitive function (*N* = 10,561).

Variables	Moderation effect of social engagement	Moderation effect of all activities
*Hearing status (ref = poor hearing)*		
Fair hearing	0.048^∗∗^ ^a^	0.051^∗∗^
	(0.017)	(0.019)
	0.004^b^	0.006

Good hearing	0.066^∗∗∗^	0.061^∗∗^
	(0.017)	(0.019)
	0.000	0.001

Very good hearing	0.061^∗∗^	0.053^∗^
	(0.020)	(0.022)
	0.002	0.015

Excellent hearing	0.017	0.030
	(0.022)	(0.025)
	0.445	0.222

Using a hearing aid	−0.012	−0.004
	(0.018)	(0.017)
	0.504	0.833

Social engagement or activity engagement	0.013^∗∗∗^	0.005^∗∗∗^
	(0.002)	(0.001)
	0.000	0.000

*Hearing X social engagement (ref = poor hearing and social engagement*)		
Fair hearing and social engagement	−0.005^∗^	−0.001
	(0.002)	(0.001)
	0.019	0.157

Good hearing and social engagement	−0.006^∗∗^	−0.001
	(0.002)	(0.001)
	0.003	0.111

Very good hearing and social engagement	−0.005^∗^	−0.001
	(0.002)	(0.001)
	0.011	0.354

Excellent hearing and social engagement	−0.003	−0.001
	(0.002)	(0.001)
	0.113	0.327

*Education (ref = 0)*		
< Elementary school	0.367^∗∗∗^	0.356^∗∗∗^
	(0.013)	(0.012)
	0.000	0.000

Elementary school+	0.670^∗∗∗^	0.641^∗∗∗^
	(0.013)	(0.012)
	0.000	0.000

*Income (ref = low)*		
Medium	0.016^∗∗^	0.013^∗^
	(0.006)	(0.006)
	0.008	0.025

High	0.053^∗∗∗^	0.052^∗∗∗^
	(0.007)	(0.006)
	0.000	0.000

Urban residence (ref = rural)	0.118^∗∗∗^	0.110^∗∗∗^
	(0.008)	(0.008)
	0.000	0.000

Female (ref = male)	0.017^∗^	−0.020^∗^
	(0.008)	(0.008)
	0.040	0.011

Age at baseline	−0.025^∗∗∗^	−0.026^∗∗∗^
	(0.000)	(0.000)
	0.000	0.000

*Random effects*		
Intercept (ln)	−1.025^∗∗∗^	−1.035^∗∗∗^
	(0.010)	(0.009)
	0.000	0.000

SE	−1.199^∗∗∗^	−1.196^∗∗∗^
	(0.006)	(0.006)
	0.000	0.000

Observations	10,561	12,601^c^

Person‐years	26,396	29,024

Chi‐square	11,071.78	−15,221

Log‐likelihood	−13,635	14,781

^a^Coefficient, standard errors in parentheses.

^b^ *p* value.

^c^The sample size changed when all activities were included.

^∗^ *p* < 0.05.

^∗∗^ *p* < 0.01.

^∗∗∗^ *p* < 0.001.

### 3.1. Robustness Tests

To assess the potential influence of selective attrition, inverse probability weighting (IPW) was implemented as a sensitivity analysis. First, respondents included in the analytic sample were identified using the estimation sample from the primary mixed‐effects model. A retention indicator was then created to identify respondents who remained under observation across all analytic waves (2015, 2018, and 2021). Next, a logistic regression model was estimated at baseline to predict the probability of remaining in the study using baseline cognitive functioning and covariates included in the primary analyses. Predicted probabilities from this model were used to generate inverse probability weights, calculated as the inverse of the estimated probability of study retention. Stabilized weights were subsequently derived to reduce variability in the weights and improve estimation efficiency. The stabilized weights were merged back into the longitudinal dataset and incorporated into weighted mixed‐effects models as probability weights. To minimize the influence of extreme weights, trimmed stabilized weights based on the 1st and 99th percentiles were used in sensitivity analyses. The patterns of mediation and moderation effects remained after adjusting for attrition (Tables 4 and 5).

**TABLE 4:** Robust test with inverse probability weights (IPWs) for attrition adjustment of mediation effects.

	Adjusted	Social engagement		All activities	
	Hearing ⟶ cognition	Hearing ⟶ social engagement	Hearing and social engagement ⟶ cognition	Hearing ⟶ social engagement	Hearing and social engagement ⟶ cognition
*Hearing status at t-1 (ref = poor hearing)*					
Fair	0.020^a^	−0.008		0.013	
	(0.019)	(0.039)		(0.034)	
	0.269^b^	0.846		0.708	

Good	0.029	0.048		0.039	
	(0.019)	(0.039)		(0.034)	
	0.120	0.218		0.253	

Very good	0.027	0.001		0.062	
	(0.021)	(0.042)		(0.038)	
	0.187	0.989		0.103	

Excellent	0.038	−0.012		0.047	
	(0.023)	(0.044)		(0.039)	
	0.100	0.778		0.237	

Using hearing aid(s)	−0.032	−0.045	−0.021	−0.092^∗^	−0.033
	(0.030)	(0.045)	(0.034)	(0.045)	
	0.281	0.322	0.543	0.042	

*Hearing status at t-2 (ref = poor hearing)*					
Fair			0.012		0.010
			(0.020)		(0.020)
			0.547		0.605

Good			0.012		0.011
			(0.020)		(0.020)
			0.543		0.605

Very good			0.020		0.017
			(0.022)		(0.022)
			0.377		0.447

Excellent			0.029		0.027
			(0.025)		(0.025)
			0.231		0.281

Social/activities engagement at t‐1			0.002^∗∗∗^		0.001^∗∗∗^
			(0.000)		(0.000)
			0.000		0.000

*Education (ref = no education)*					
Incomplete elementary school	0.354^∗∗∗^	0.323^∗∗∗^	0.370^∗∗∗^	0.404^∗∗∗^	0.367^∗∗∗^
	(0.017)	(0.040)	(0.017)	(0.027)	(0.017)
	0.000	0.000	0.000	0.000	0.000

Complete elementary school and upper	0.682^∗∗∗^	0.775^∗∗∗^	0.695^∗∗∗^	0.801^∗∗∗^	0.689^∗∗∗^
	(0.016)	(0.038)	(0.017)	(0.026)	(0.017)
	0.000	0.000	0.000	0.000	0.000

*Income (ref = low)*					
Medium income	0.015	0.093^∗∗∗^	0.009	0.093^∗∗∗^	0.010
	(0.009)	(0.018)	(0.009)	(0.015)	(0.009)
	0.079	0.000	0.313	0.000	0.285

High income	0.065^∗∗∗^	0.226^∗∗∗^	0.060^∗∗∗^	0.180^∗∗∗^	0.062^∗∗∗^
	(0.009)	(0.020)	(0.010)	(0.016)	(0.010)
	0.000	0.000	0.000	0.000	0.000

Urban residence	0.124^∗∗∗^	0.177^∗∗∗^	0.118^∗∗∗^	0.217^∗∗∗^	0.115^∗∗∗^
	(0.009)	(0.023)	(0.010)	(0.016)	(0.010)
	0.000	0.000	0.000	0.000	0.000

Female	0.017	0.194^∗∗∗^	0.009	0.325^∗∗∗^	0.005
	(0.009)	(0.023)	(0.010)	(0.015)	(0.010)
	0.073	0.000	0.352	0.000	0.592

Age at baseline	−0.026^∗∗∗^	−0.022^∗∗∗^	−0.028^∗∗∗^	−0.018^∗∗∗^	−0.028^∗∗∗^
	(0.001)	(0.001)	(0.001)	(0.001)	(0.001)
	0.000	0.000	0.000	0.000	0.000

*Random effects*					
SE of random intercept (ln)	−0.924^∗∗∗^		−0.864^∗∗∗^		−0.868^∗∗∗^
	(0.011)		(0.011)		(0.011)
	0.000		0.000		0.000

SE of residual error (ln)	−1.261^∗∗∗^		−1.387^∗∗∗^		−1.386^∗∗∗^
	(0.013)		(0.016)		(0.016)
	0.000		0.000		0.000

Overdispersion (ln)		−1.631^∗∗∗^		−1.571^∗∗∗^	
		(0.038)		(0.033)	
		0.000		0.000	

SE of the random intercept		0.932^∗∗∗^		0.995^∗∗∗^	
		(0.024)		(0.023)	
		0.000		0.000	

Person‐years	21,851	21,851	18,333	21,850	18,332

Number of observations	9995	9995	9200	9995	9199

Log‐likelihood	−12,502	−69,792	−10,671	−119,654	−10,654

Chi‐square	7023	1575	7217	3466	7258

^a^Coefficient, standard errors in parentheses.

^b^ *p* value.

^∗^ *p* < 0.05.

^∗∗^ *p* < 0.01.

^∗∗∗^ *p* < 0.001.

**TABLE 5:** Robust test with inverse probability weights (IPWs) for attrition adjustment of moderation effects.

Variables	Moderation effect with social engagement	Moderation effect with all activities
*Hearing status (ref = poor hearing)*		
Fair hearing	0.032^a^	0.028
	(0.024)	(0.028)
	0.195^b^	0.318

Good hearing	0.042	0.034
	(0.024)	(0.028)
	0.080	0.225

Very good hearing	0.046	0.044
	(0.027)	(0.032)
	0.090	0.178

Excellent hearing	−0.013	−0.008
	(0.030)	(0.036)
	0.674	0.832

Using a hearing aid	0.007	0.003
	(0.028)	(0.027)
	0.806	0.899

Activities (0‐50)	0.010^∗∗∗^	0.004^∗∗∗^
	(0.002)	(0.001)
	0.000	0.000

*Hearing X social engagement (ref = poor hearing and social engagement)*		
Fair hearing and social engagement	−0.002	−0.000
	(0.002)	(0.001)
	0.302	0.647

Good hearing and social engagement	−0.003	−0.000
	(0.002)	(0.001)
	0.147	0.567

Very good hearing and social engagement	−0.003	−0.000
	(0.002)	(0.001)
	0.248	0.628

Excellent hearing and social engagement	−0.001	0.000
	(0.002)	(0.001)
	0.773	0.995

*Education (ref = 0)*		
< Elementary school	0.380^∗∗∗^	0.371^∗∗∗^
	(0.016)	(0.015)
	0.000	0.000

Elementary school+	0.687^∗∗∗^	0.669^∗∗∗^
	(0.016)	(0.015)
	0.000	0.000

*Income (ref = low)*		
Medium	0.016^∗^	0.005
	(0.008)	(0.008)
	0.039	0.517

High	0.046^∗∗∗^	0.029^∗∗^
	(0.008)	(0.009)
	0.000	0.001

Urban residence (ref = rural)	0.122^∗∗∗^	0.118^∗∗∗^
	(0.009)	(0.009)
	0.000	0.000

Female (ref = male)	0.014	−0.020^∗^
	(0.009)	(0.008)
	0.126	0.017

Age at baseline	−0.026^∗∗∗^	−0.027^∗∗∗^
	(0.001)	(0.001)
	0.000	0.000

*Random effects*		
Intercept (ln)	−0.929^∗∗∗^	−0.880^∗∗∗^
	(0.011)	(0.009)
	0.000	0.000

SE	−1.240^∗∗∗^	−1.386^∗∗∗^
	(0.011)	(0.011)
	0.000	0.000

Observations	10,561	12,601^c^

Person‐years	26,396	29,024

Chi‐square	−15,035	−15,602

Log‐likelihood	8786	10,973

^a^Coefficient, standard errors in parentheses.

^b^ *p* value.

^c^The sample size changed when all activities were included.

^∗^ *p* < 0.05.

^∗∗^ *p* < 0.01.

^∗∗∗^ *p* < 0.001.

To test the robustness of the results, we re‐estimated the models excluding those who were using hearing aids during the survey. The pattern between hearing status and cognitive function changed slightly (the connection between good hearing and cognitive function was attenuated), whereas the link between hearing status and social engagement did not change (Appendix Table A4). Second, we re‐estimated all models with the original highest educational attainment (no education, incomplete elementary school, elementary school, middle school, high school, and college or higher). The pattern changed slightly but largely remained (Appendix Table A5). In addition, considering the correlations between self‐reported hearing status and education, we also examined the interaction effects of hearing status and education, and the interaction effects of social engagement and education (Appendix Table A6). The results showed that participants with high school education and fair hearing, or indeed high school education and very good hearing, had lower cognition (*b* = −0.223, SE = 0.074, *p* = 0.003 and *b* = −0.163, SE = 0.082, *p* = 0.045, respectively). It is possible that those with high school education include a high level of heterogeneity. Moreover, participants with any level of education showed lower cognition with a higher level of social engagement. One explanation is that social engagement is more likely to have protective effects for those with no education.

## 4. Discussion

This study evaluated the influence of social engagement on the longitudinal association between hearing status and cognitive function in middle‐aged and older adults living in Mexico. The present findings demonstrate a positive relationship between hearing status and cognitive functioning, in alignment with the findings of similar studies (e.g., [37, 38]). By introducing lagging variables, we found that better hearing status was longitudinally associated with higher levels of social engagement and better global cognition in the following survey wave in unadjusted models. However, the effect size is quite modest. There is a significant association between hearing status at baseline, social engagement at the following wave, and cognitive function in the later wave in the unadjusted model. However, this association was attenuated when adjusted for social engagement and all covariates. Previous studies indicate that Mexico, as well as many other Latin American countries, exhibits higher population attributable fractions (PAFs) for dementia than global averages due to the greater prevalence of modifiable risk factors such as lower educational attainment and health‐risk behaviors [39, 40]. For example, the PAF of education for the global population is 5% [41], but it is 11% in Latin America [39] due to the universally low level of education among older adults. The PAF of hearing loss is also higher in Latin America, as a high proportion of the population has untreated hearing loss. These contextual conditions may influence the extent to which hearing difficulty contributes to cognitive decline, confounding the socioeconomic settings [42]. Another potential explanation is that individuals have established social routines and networks, and a deterioration in hearing status did not significantly alter their social engagement (De Bruin et al., 2021), which may explain the nonsignificant mediation effects of social engagement on the association between hearing status and cognitive functioning.

Second, we identified positive impacts of social engagement on cognitive functioning among middle‐aged and older adults in Mexico. This finding aligns with previous research suggesting that maintaining social ties and participating in social engagement can act as a buffer against cognitive decline [10]. However, our study did not find a significant association between hearing status in the previous survey wave and social engagement in the adjusted model, which contrasts with existing literature suggesting that hearing impairment often limits social participation [11]. For instance, in a study of 2000 older adults enrolled in the Health, Well‐being, and Aging Study, those with self‐rated poor hearing at baseline had a significantly greater decline in social participation scores over 9 years compared to those with self‐rated good hearing [43]. Similarly, in a study of 760 community‐dwelling older adults enrolled in the Life‐Space Mobility in Old Age project, those who reported major hearing difficulty at baseline had significantly greater odds of withdrawal from any leisure activity over 2 years of follow‐up compared to those who reported no major hearing difficulty [44]. These associations have logical mechanistic underpinnings, as the loss of auditory input resulting from hearing loss is likely to discourage participation in social engagement, which, in turn, may dilute the protective effects of social interaction on cognition [45]. Our findings suggest that hearing status may be an antecedent of engagement in social engagement, but education, income, and demographic characteristics contribute to the link between hearing status and social engagement. This assertion aligns with the findings of a recent systematic review and meta‐analysis of 15 longitudinal studies, which similarly found no mediation by social isolation on the longitudinal association between hearing loss and subsequent cognition in adults [46].

The distinct cultural context of Mexico presents an opportunity to evaluate a conceptual framework among a cohort of older adults whose social structures and life‐course experiences differ markedly from those of the US populations in whom the theories of our conceptual framework were originally developed. For instance, Mexican culture is characterized by familismo (i.e., strong obligations to and reliance on family networks), a central feature of social life among Mexican older adults [20], and this, in turn, may shape how social engagement confers cognitive benefits. Prior research using MHAS data found that among married couples in Mexico, a wife’s social engagement benefited the cognitive function of both spouses, whereas the husband’s social engagement was unrelated to the wife’s cognition [34]. Such asymmetry could be attributed to more traditional gendered social roles in Mexico, contrasting with contemporary patterns observed in the US. This suggests the cultural context of social engagement is an important consideration, which provides justification for evaluating our research questions in adults aged 50 and above in Mexico.

Finally, the results from the moderation analysis suggest that the relationship between hearing status and cognitive functioning differs according to levels of social engagement. We found that participants with fair to very good hearing and high social engagement had lower cognitive functioning than participants with poor hearing. These findings suggest that participants with poor hearing may experience greater cognitive benefits from social engagement than participants with fair to very good hearing. This finding is inconsistent with previous studies [47–49]. One possibility is that the measure of hearing status in this study is based on self‐reported evaluations, which may not effectively distinguish between severe hearing loss and mild hearing difficulty.

Several factors may explain the modest effect sizes observed in this study. First, cognitive functioning is influenced by numerous biological, social, and environmental determinants, making it unlikely that perceived hearing status alone would account for a substantial proportion of cognitive variation. Second, the pathway linking hearing difficulties to cognition through social participation is likely indirect and subject to considerable heterogeneity, as many individuals maintain social engagement despite hearing challenges through adaptation and support. Third, adjustment for a broad set of demographic and socioeconomic covariates may appropriately reduce the magnitude of the association by accounting for factors that are shared determinants of hearing, social engagement, and cognition. Moreover, the findings are most appropriately interpreted as evidence of a population‐level association rather than a clinically significant difference in cognitive functioning for any individual participant.

Our findings must be interpreted in the context of several limitations. First, self‐reported hearing status could have introduced misclassification bias and potentially diluted our exposure–outcome associations. However, while objective (e.g., audiometric) assessments would provide more precise estimates of hearing status, they represent an impractical method of evaluating hearing status at a population level, and the use of self‐reported measures in the present study aligns with the approach used in previous studies (see [50]). Second, the final sample is healthy‐biased by excluding the proxy respondents who are more vulnerable, older, and with worse health. The pattern of the missing data also strengthened this healthy bias. To be specific, participants with lower cognitive function, poor hearing, less social engagement, and older age are more likely to have missing data, which may lead to underestimation of the impacts of hearing difficulty and social engagement on global cognition. Third, the low prevalence of hearing aid use in the analytic sample means we cannot generalize to a wider older adult Mexican population, among whom the prevalence of hearing aid use is 3.0%, which limits the ability to examine how auditory interventions may impact cognitive outcomes or social engagement. Finally, because sampling weights were not incorporated into the mixed‐effects models, the findings should be interpreted as associations observed within the analytic sample rather than as nationally representative estimates for older adults in Mexico.

## 5. Conclusion

This study explores the complex interplay between hearing status, social engagement, and cognitive function in Mexico’s context. The findings suggest that better hearing status is associated with better cognitive function over time and highlight the positive influence of social engagement on both hearing status and cognitive function, while the effect sizes are quite modest. On the other hand, however, these links are attenuated after adjusting for covariates, which suggests that the links were affected by demographic characteristics and socioeconomic status in Mexico. As for the moderation effect, we find that social engagement benefits more for participants with poor hearing.

## Funding

This study was supported by the National Institute on Aging (Grant nos. R01AG068988, R01AG018016, T32AG000270, P30AG059301, and P30AG024832).

## Conflicts of Interest

The authors declare no conflicts of interest.

## Data Availability

The data used in this paper are publicly accessible at the following websites: https://www.mhasweb.org/Home/index.aspx, https://www.mhasweb.org/DataProducts/Home.aspx, and https://www.mhasweb.org/DataProducts/CoreSurveyData.aspx.

## Appendix A

**TABLE A1:** Characteristics of missing data (person‐years).

Variable	Missing		Included	
	Observations	Mean (SD)	Observations	Mean (SD)
Cognition (*z*‐score)	3064	0.03 (0.072)	26,396	0.07 (0.60)
Hearing status				
Poor	6184	6.95%	26,396	3.94%
Fair	6184	32.23%	26,396	32.17%
Good	6184	46.10%	26,396	48.06%
Very good	6184	9.61%	26,396	10.18%
Excellent	6184	5.11%	26,396	5.65%
Using a hearing aid	7824	3.27%	26,396	2.19%
Social engagement (0–50)	4704	3.20 (5.46)	26,396	8.02 (8.27)
Education				
No education	14,677	21.01%	26,396	13.49%
< 6 years	14,677	28.31%	26,396	29.99%
≥ 6 years	14,677	50.68%	26,396	56.52%
Income				
Low	8087	35.71%	26,396	31.52%
Medium	8087	34.90%	26,396	35.08%
High	8087	29.39%	26,396	33.40%
Urban residence	15,154	58.27%	26,396	57.24%
Sex				
Man	13,468	46.01%	26,396	40.83%
Women	13,468	53.99%	26,396	59.17%
Age at baseline	13,468	69.25 (10.51)	26,396	65.10 (8.59)

**TABLE A2:** Characteristics of missing data at baseline.

Variable	Missing		Included	
	Observations	Mean (SD)	Observations	Mean (SD)
Cognition (*z*‐score)	2210	−0.02 (0.76)	10,145	0.05 (0.64)
Hearing status				
Poor	3100	6.03%	10,145	3.76%
Fair	3100	30.71%	10,145	30.32%
Good	3100	49.26%	10,145	51.89%
Very good	3100	9.32%	10,145	9.42%
Excellent	3100	4.68%	10,145	4.61%
Using a hearing aid	3140	1.97%	10,145	1.72%
Social engagement (0–50)	810	4.25 (6.18)	10,145	8.21 (8.80)
Education				
No education	3546	20.56%	10,145	14.65%
< 6 years	3546	26.20%	10,145	30.51%
≥ 6 years	3546	53.24%	10,145	54.84%
Income				
Low	3655	35.73%	10,145	32.25%
Medium	3655	33.05%	10,145	33.87%
High	3655	31.22%	10,145	33.88%
Urban residence	3705	60.38%	10,145	56.61%
Sex				
Man	3143	46.07%	10,145	41.50%
Women	3143	53.93%	10,145	58.50%
Age at baseline	3143	68.56 (10.68)	10,145	65.87 (8.99)

**TABLE A3:** The range of each cognitive task.

Cognitive task	Original range	Range of *z*‐score
Verbal learning (immediate recall)	0–8	−3.552, 2.492
Verbal recall (delayed recall)	0–8	−1.938, 1.798
Figure copy	0–6	−5.008, 0.432
Figure recall	0–6	−2.631, 0.718
Verbal fluency	0–40	−2.903, 4.641
Visual scanning	0–60	−1.781, 1.870
Orientation	0–3	−2.805, 0.664

**TABLE A4:** Mixed‐effects models of lagged hearing and activity engagement predicting cognitive function, excluding hearing aid(s).

	Hearing ⟶ cognition	Hearing ⟶ social engagement	Hearing and social engagement ⟶ cognition
*Hearing status at t-1 (ref = poor hearing)*			
Fair	0.022^a^	0.002	
	(0.015)	(0.034)	
	0.147^b^	0.953	

Good	0.027	0.049	
	(0.015)	(0.034)	
	0.064	0.155	

Very good	0.037^∗^	0.005	
	(0.016)	(0.038)	
	0.023	0.892	

Excellent	0.041^∗^	−0.010	
	(0.018)	(0.041)	
	0.023	0.800	

*Income (ref = low)*			
Medium income	0.017^∗∗^	0.096^∗∗∗^	0.014
	(0.007)	(0.015)	(0.007)
	0.009	0.000	0.051

High income	0.073^∗∗∗^	0.243^∗∗∗^	0.072^∗∗∗^
	(0.008)	(0.017)	(0.008)
	0.000	0.000	0.000

Urban residence	0.121^∗∗∗^	0.170^∗∗∗^	0.111^∗∗∗^
	(0.009)	(0.021)	(0.010)
	0.000	0.000	0.000

Female	0.021^∗^	0.175^∗∗∗^	0.021^∗^
	(0.009)	(0.021)	(0.009)
	0.016	0.000	0.027

Age at baseline	−0.025^∗∗∗^	−0.017^∗∗∗^	−0.026^∗∗∗^
	(0.001)	(0.001)	(0.001)
	0.000	0.000	0.000

*Hearing status at t-2 (ref = poor hearing*)			
Fair			0.021
			(0.016)
			0.189

Good			0.018
			(0.016)
			0.243

Very good			0.027
			(0.018)
			0.118

Excellent			0.034
			(0.019)

*Education (ref = no education)*			0.074
Incomplete elementary school	0.346^∗∗∗^	0.231^∗∗∗^	0.353^∗∗∗^
	(0.014)	(0.033)	(0.014)
	0.000	0.000	0.000

Complete elementary school and upper	0.673^∗∗∗^	0.667^∗∗∗^	0.671^∗∗∗^
	(0.013)	(0.032)	(0.014)
	0.000	0.000	0.000

Social engagement at t‐1			0.004^∗∗∗^
			(0.000)
			0.000

Constant	1.078^∗∗∗^	2.012^∗∗∗^	1.072^∗∗∗^
	(0.041)	(0.098)	(0.044)
	0.000	0.000	0.000

*Random effects*			
SE of random intercept (ln)	−1.009^∗∗∗^		−1.020^∗∗∗^
	(0.010)		(0.011)
	0.000		0.000

SE of residual error (ln)	−1.216^∗∗∗^		−1.207^∗∗∗^
	(0.007)		(0.008)
	0.000		0.000

Overdispersion (ln)		−1.463^∗∗∗^	
		(0.024)	
		0.000	

SE of the random intercept		0.729^∗∗∗^	
		(0.017)	
		0.000	

Person‐years	21,369	21,369	17,923

Number of observations	9873	9873	9065

Log‐likelihood	−11,309	−63,873	−9670

Chi‐square	8430	1631	7799

^a^Coefficient, standard errors in parentheses.

^b^ *p* value.

^∗^ *p* < 0.05.

^∗∗^ *p* < 0.01.

^∗∗∗^ *p* < 0.001.

**TABLE A5:** Sensitivity test with original educational attainments.

	Hearing ⟶ cognition	Hearing ⟶ social engagement	Hearing and social engagement ⟶ cognition
*Hearing status at t-1 (ref = poor hearing)*			
Fair	0.027^a^	0.005	
	(0.014)	(0.033)	
	0.060^b^	0.883	

Good	0.025	0.039	
	(0.014)	(0.033)	
	0.073	0.235	

Very good	0.030	−0.009	
	(0.016)	(0.036)	
	0.062	0.813	

Excellent	0.037^∗^	−0.017	
	(0.017)	(0.039)	
	0.034	0.663	

Using hearing aid(s)	−0.034	−0.059	−0.038
	(0.019)	(0.044)	(0.021)
	0.081	0.176	0.073

*Education (ref = no education)*			
Incomplete elementary school	0.351^∗∗∗^	0.242^∗∗∗^	0.359^∗∗∗^
	(0.013)	(0.032)	(0.014)
	0.000	0.000	0.000

Complete elementary school	0.548^∗∗∗^	0.451^∗∗∗^	0.553^∗∗∗^
	(0.014)	(0.034)	(0.015)
	0.000	0.000	0.000

Middle school	0.737^∗∗∗^	0.740^∗∗∗^	0.741^∗∗∗^
	(0.015)	(0.037)	(0.016)
	0.000	0.000	0.000

High school	0.809^∗∗∗^	0.907^∗∗∗^	0.788^∗∗∗^
	(0.023)	(0.053)	(0.024)
	0.000	0.000	0.000

College and higher	0.927^∗∗∗^	1.145^∗∗∗^	0.921^∗∗∗^
	(0.018)	(0.044)	(0.020)
	0.000	0.000	0.000

*Income (ref = low)*			
Medium income	0.015^∗^	0.093^∗∗∗^	0.011
	(0.007)	(0.015)	(0.007)
	0.024	0.000	0.127

High income	0.046^∗∗∗^	0.199^∗∗∗^	0.044^∗∗∗^
	(0.007)	(0.017)	(0.008)
	0.000	0.000	0.000

Urban residence	0.088^∗∗∗^	0.108^∗∗∗^	0.081^∗∗∗^
	(0.009)	(0.021)	(0.009)
	0.000	0.000	0.000

Female	0.036^∗∗∗^	0.209^∗∗∗^	0.033^∗∗∗^
	(0.009)	(0.020)	(0.009)
	0.000	0.000	0.000

Age at baseline	−0.024^∗∗∗^	−0.015^∗∗∗^	−0.025^∗∗∗^
	(0.001)	(0.001)	(0.001)
	0.000	0.000	0.000

*Hearing status at t-2 (ref = poor hearing)*			
Fair			0.023
			(0.015)
			0.126

Good			0.015
			(0.015)
			0.236

Very good			0.021
			(0.017)
			0.207

Excellent			0.028
			(0.019)
			0.131

Social engagement at t‐1			0.003^∗∗∗^
			(0.000)
			0.000

Constant	1.008^∗∗∗^	1.878^∗∗∗^	1.022^∗∗∗^
	(0.040)	(0.095)	(0.042)
	0.000	0.000	0.000

*Random effects*			
SE of random intercept (ln)	−1.050^∗∗∗^		−1.052^∗∗∗^
	(0.010)		(0.011)
	0.000		0.000

SE of residual error (ln)	−1.212^∗∗∗^		−1.207^∗∗∗^
	(0.007)		(0.008)
	0.000		0.000

Overdispersion (ln)		−1.464^∗∗∗^	
		(0.023)	
		0.000	

SE of the random intercept		0.690^∗∗∗^	
		(0.016)	
		0.000	

Person‐years	21,851	21,851	18,333

Number of observations	9995	9995	9200

Log‐likelihood	−11,285	−65,064	−9665

Chi‐square	9712	2071	8835

^a^Coefficient, standard errors in parentheses.

^b^ *p* value.

^∗^ *p* < 0.05.

^∗∗^ *p* < 0.01.

^∗∗∗^ *p* < 0.001.

**TABLE A6:** The moderation effects of education.

	Model 1 hearing X education	Model 2 social engagement X education
*Hearing status (ref = poor hearing)*		
Fair hearing	0.076^∗∗^ ^a^	0.026^∗^
	(0.027)	(0.013)
	0.005^b^	0.045

Good hearing	0.034	0.028^∗^
	(0.028)	(0.013)
	0.228	0.036

Very good hearing	0.035	0.024
	(0.034)	(0.015)
	0.306	0.101

Excellent hearing	0.006	−0.001
	(0.041)	(0.016)
	0.890	0.940

Using a hearing aid	−0.015	−0.015
	(0.018)	(0.018)
	0.381	0.381

Social engagement (0‐50)	0.007^∗∗∗^	0.011^∗∗∗^
	(0.000)	(0.001)
	0.000	0.000

*Hearing X education (ref = poor hearing/no education)*		
Fair_#incomplete elementary school	−0.052	
	(0.035)	
	0.135	

Fair_#elementary school	−0.038	
	(0.040)	
	0.333	

Fair_#middle school	−0.083	
	(0.045)	
	0.066	

Fair_#high school	−0.223^∗∗^	
	(0.074)	
	0.003	

Fair_#college and higher	−0.042	
	(0.073)	
	0.565	

Good_#incomplete elementary school	−0.006	
	(0.035)	
	0.871	

Good_#elementary school	0.003	
	(0.041)	
	0.944	

Good_#middle school	−0.018	
	(0.046)	
	0.696	

Good_#high school	−0.137	
	(0.074)	
	0.065	

Good_#college and higher	0.012	
	(0.073)	
	0.870	

Very good_#incomplete elementary school	−0.011	
	(0.043)	
	0.787	

Very good_#elementary school	0.000	
	(0.047)	
	0.999	

Very good_#middle school	−0.014	
	(0.052)	
	0.782	

Very good_#high school	−0.163^∗^	
	(0.082)	
	0.045	

Very good_#college and higher	0.005	
	(0.077)	

Excellent_#incomplete elementary school	−0.008	
	(0.050)	
	0.568	

Excellent_#elementary school	−0.031	
	(0.055)	
	0.679	

Excellent_#middle school	−0.024	
	(0.058)	
	0.448	

Excellent_#high school	−0.068	
	(0.090)	

Excellent_#college and higher	0.039	
	(0.083)	
	0.637	

*Education (ref = no education)*		
Incomplete elementary school	0.396^∗∗∗^	0.384^∗∗∗^
	(0.034)	(0.014)
	0.000	0.000

Complete elementary school	0.573^∗∗∗^	0.581^∗∗∗^
	(0.039)	(0.016)
	0.000	0.000

Middle school	0.780^∗∗∗^	0.768^∗∗∗^
	(0.045)	(0.017)
	0.000	0.000

High school	0.952^∗∗∗^	0.825^∗∗∗^
	(0.073)	(0.027)
	0.000	0.000

College and higher	0.905^∗∗∗^	0.938^∗∗∗^
	(0.072)	(0.022)
	0.000	0.000

*Income (ref = low)*		
Medium income	0.015^∗^	0.015^∗^
	(0.006)	(0.006)
	0.012	

High income	0.035^∗∗∗^	0.034^∗∗∗^
	(0.007)	(0.007)
	0.000	0.000

Urban residence	0.090^∗∗∗^	0.090^∗∗∗^
	(0.008)	(0.008)
	0.000	0.000

Female	0.032^∗∗∗^	0.032^∗∗∗^
	(0.008)	(0.008)
	0.000	0.000

Age at baseline	−0.024^∗∗∗^	−0.024^∗∗∗^
	(0.000)	(0.000)
	0.000	0.000

*Social engagement X education*		
Incomplete elementary school#social engagement		−0.003
		(0.002)
		0.090

Elementary school#social engagement		−0.005^∗∗^
		(0.002)
		0.003

Middle school#social engagement		−0.005^∗∗^
		(0.002)
		0.001

High school#social engagement		−0.005^∗∗^
		(0.002)
		0.005

College and higher#social engagement		−0.005^∗∗∗^
		(0.002)
		0.001

Constant	0.923^∗∗∗^	0.919^∗∗∗^
	(0.044)	(0.039)
	0.000	0.000

*Random effects*		
SE of random intercept (ln)	−1.057^∗∗∗^	−1.058^∗∗∗^
	(0.010)	(0.010)
	0.000	0.000

SE of residual error (ln)	−1.201^∗∗∗^	−1.200^∗∗∗^
	(0.006)	(0.006)
	0.000	0.000

Person‐years	26,396	26,396

Number of observations	10,561	10,561

Log‐likelihood	−13,361	−13,366

Chi‐square	12,156	12,160

^a^Coefficient, standard errors in parentheses.

^b^ *p* value.

^∗^ *p* < 0.05.

^∗∗^ *p* < 0.01.

^∗∗∗^ *p* < 0.001.
